# The Impact of 9-Valent HPV Vaccination on Couple Infertility Prevention: A Comprehensive Review

**DOI:** 10.3389/fmed.2021.700792

**Published:** 2021-08-17

**Authors:** Andrea Ciavattini, Chiara Marconi, Luca Giannella, Giovanni Delli Carpini, Francesco Sopracordevole, Jacopo Di Giuseppe

**Affiliations:** ^1^Woman's Health Sciences Department, Gynecologic Section, Polytechnic University of Marche, Ancona, Italy; ^2^Gynecological Oncology Unit, Istituto di Ricovero e Cura a Carattere Scientifico Centro di Riferimento Oncologico (IRCCS CRO) Centro di Riferimento Oncologico – National Cancer Institute, Aviano, Italy

**Keywords:** infertility, human papillomavirus, vaccine, assisted reproduction (ART), miscarriage

## Abstract

A comprehensive literature review was performed to determine the relationship between HPV infection and infertility and the eventual role of the 9-valent vaccine for infertility prevention. The search was extended from January 1997 through July 2021. Data collected from selected articles focused on three main topics: statistical associations between HPV prevalence and assisted reproductive technology (ART) outcome, association between HPV and characteristics of semen, and associations between HPV and miscarriage. Articles that identified HPV genotypes were selected for this review to study the possible role of the 9-valent vaccine in infertility prevention. To date, there is no agreement on the implication HPV female infection has on the fertility and miscarriage rate. Although it can be stated that HPV prevalence among couples with infertility undergoing ART treatment is consistent, it does not seem to affect the performance of oocytes. Otherwise, HPV infection affects sperm parameters, in particular spermatozoa motility. When an association can be found, most cases of HR-HPV involved are those included in the 9-valent vaccine. The correlation between HPV male infection both with asthenozoospermia and increased risk of pregnancy loss could recommend the extension of anti-HPV vaccination to adolescent males along with cancer prevention. Despite the fact that the relation between 9-valent HPV genotypes involved in female infection and miscarriage/infertility is not clear, the impact of this virus on health reproduction is evident. Considering this, the importance of HPV vaccination in adolescent females is confirmed. A vaccine efficacy study could be useful to confirm the importance of primary prevention for couple reproductive health.

## Introduction

Infertility is a complex human health situation that particularly alters the quality of life in couples that face it. It represents a worldwide problem that affects about 10–30% of couples of reproductive ages (1, 2). The American College of Obstetricians and Gynaecologists (ACOG) defines infertility as the failure to achieve pregnancy within 12 months of unprotected intercourse or therapeutic donor insemination in women younger than 35 years or within 6 months in women older than 35 years (3).

Worldwide, the primary cause of infertility is sexually transmitted diseases (STDs) (4, 5). The role of *Chlamydia trachomatis* and *Neisseria gonorrhoeae* infection in salpingitis and infertility has been widely studied as well as the possible damage caused by *Trichomonas vaginalis, Mycoplasma genitalium*, and other microorganisms within the vaginal microbiome. While early diagnosis and treatment for *C. trachomatis* and *N. gonorrhoeae* have been developed for the prevention of pelvic inflammatory disease (PID) and subsequent tubal factor infertility (TFI), additional data are needed to determine whether early detection of other potential pathogens can reduce the incidence of TFI (6).

One of those is human papillomavirus (HPV). HPV infections are significantly associated with cancer of the male and female anogenital mucosa (7, 8) and some adverse effects on reproductive functions (9, 10). Most of these consequences are caused by the inability of the immune system to spontaneously clear HPV: in particular, high-risk HPV (HR-HPV) types are more likely to persist than low-risk HPV types (11–13). The infection is often asymptomatic, and most of the time, people are infected without being aware.

Despite genital HPV infection being the most common sexually transmitted viral infection worldwide, with an estimated overall prevalence of 10% in the general female population during reproductive age (14, 15), few studies have investigated the effect of HPV infection on human reproduction.

Research over the last few years has been carried out to understand if HPV could be an etiological agent that determines infertility or miscarriage and if assisted reproductive technology (ART) requires specific management for HPV-positive patients (9).

If this correlation were real, HPV prevention would play a dual role, not only for cancer but also for infertility. However, in order to perform efficacious prevention on infertility and adequate counseling in infertile couples, it is important to assess which HPV type could cause infertility or miscarriage. Identifying the prevalent genotypes could be important also to evaluate the possible role of the vaccine in preventing HPV-related infertility. In fact, it has not been studied yet if one of the three disposable HPV vaccines could significantly provide protection against infertility as well as neoplastic diseases.

The purpose of this comprehensive review is to research the available knowledge regarding the implication of HPV genotypes included in the 9-valent vaccine HR-HPV 16, 18, 31, 33, 45, 52, and 58 and low-risk HPV (LR-HPV) 6 and 11 in couple infertility with the aim to hypothesize whether vaccination could have a protective role for reproductive health.

## Methods

**Figure 1:**
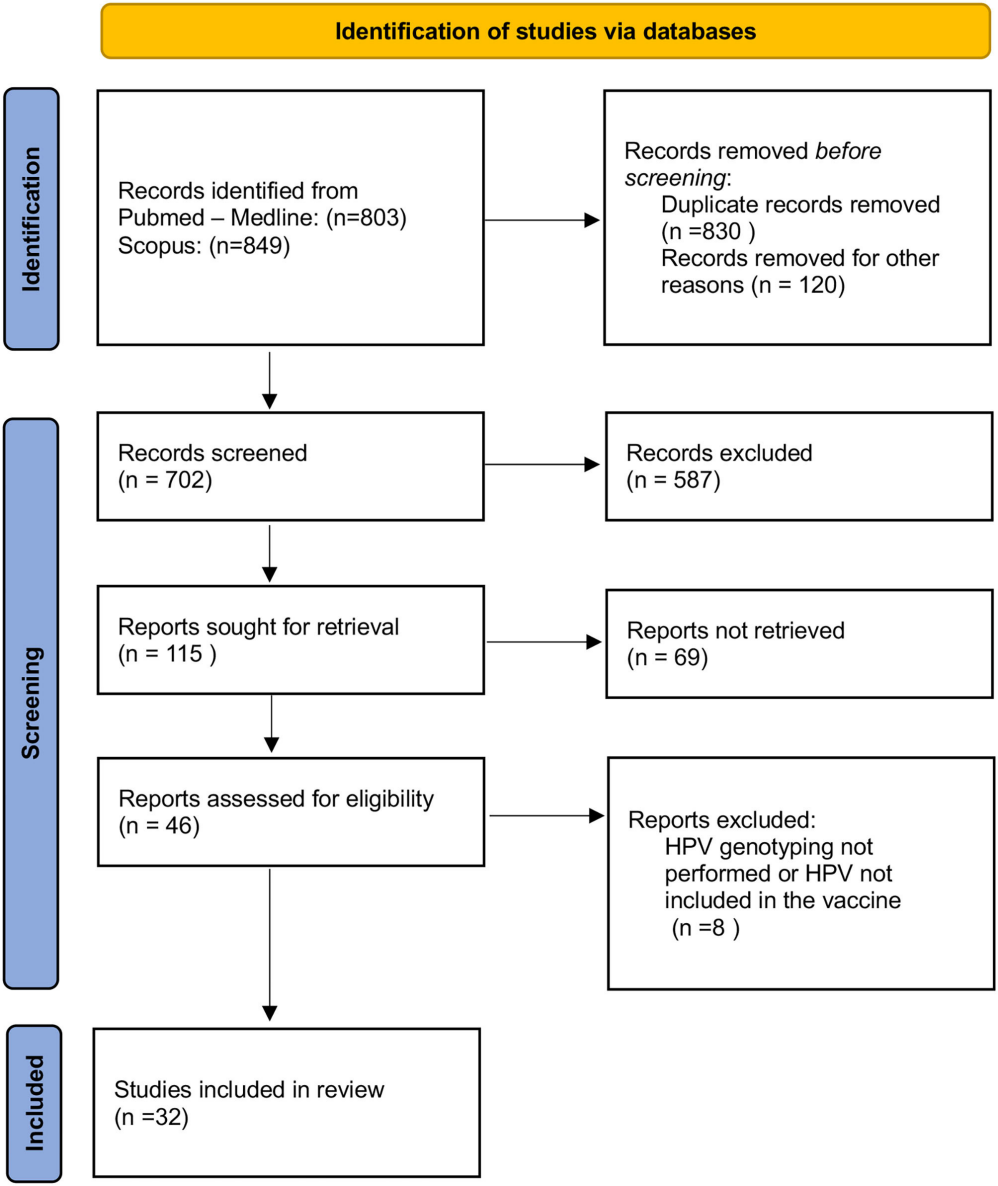
Flow chart of the literature review and study selection process.

A comprehensive literature search was performed from January 1997 through July 2021 (Figure 1). The following Mesh terms were searched: human papillomavirus, fertility, infertility, miscarriage, *in vitro* fertilization (IVF), assisted reproductive technology (ART), sperm, and blastocyst in PubMed/Medline (all fields) (last accessed on July 5, 2021) and Scopus (Title/Abstract/Keywords) (last accessed on July 5, 2021) databases. Relevant articles were selected for full-text reading and were restricted to those in the English language. Titles and abstracts were screened for relevance to determine which articles were to undergo full-text review. Articles identified as potentially relevant moved into a full-text review. The references of included studies were reviewed to identify additional publications not found through the database search.

The PRISMA (Preferred Reporting Items for Systematic Reviews and Meta-Analyses) guidelines were followed to systematically review the literature by searching the PubMed/Medline and Scopus database. The present study assessed the following PICOS (Population, Intervention, Comparison, Outcomes) questions:

- Population: couples, men, or women affected by infertility with HPV infection;
- Intervention: observe data on IVF cycles, sperm analysis, ART, and pregnancy (miscarriage) outcomes;
- Comparison: no comparisons are expected;
- Outcomes: (1) prevalence of HPV genotype infection, with particular reference to those included in the 9-valent vaccine; (2) ART, semen parameters, and pregnancy outcomes.

### Study Design

Review and meta-analysis, prospective and retrospective observational studies, population-based cohort study, prospective cohort study, case-control study, and cross-sectional clinical study.

### Eligibility/Inclusion Criteria

Retrospective as well as prospective studies that present results from studies on population-based correlations between human reproductive health and HPV. The search also retrieved articles that are based on *in vitro* experimentations on mice embryos to explain the effect of HPV infection on reproductive health (16, 17). Articles refer to almost one HPV genotype included in the 9-valent vaccine.

### Exclusion Criteria

Articles referring to HPV genotypes other than those not included in 9-valent HPV vaccines (or unspecified HPV genotypes); cases in a non-English language.

### Information Sources and Search Strategy

We searched for (HPV) AND (infertility OR fertility OR miscarriage OR *in vitro* fertilization—IVF OR assisted reproductive technology—ART OR sperm OR blastocyst) in PubMed (all fields), Medline (all fields) (accessed on July 5, 2021), and Scopus (Title/Abstract/Keywords) (accessed on July 5, 2021) databases. The only filter used was the English language. Relevant articles were obtained in full-text format and screened for additional references.

### Study Selection

An independent reviewer (C. M.) selected the studies using a two-step screening method. At first, the screening of titles and abstracts was performed to assess eligibility and inclusion criteria and exclude non-relevant studies. Afterward, the reviewer evaluated full texts of included articles to assess study eligibility and the inclusion criteria and to avoid duplications of the included cases. Then, the same author performed a manual search of reference lists to search for additional relevant publications. J. D. G. and G. D. C. checked the data extracted.

The objective of this review was to determine the relationship between HPV infection and infertility and the eventual role of the 9-valent vaccine for infertility prevention.

### Data Collection Process/Data Items

Data collection was study-related (authors and year of study publication) and case-related (HPV genotypes, IVF and pregnancy outcomes, and sperm characteristics).

The data collected from selected articles focused on three main topics:

- statistical associations between HPV genotypes included in the 9-valent vaccine and ART outcome;
- associations between HPV genotypes included in the 9-valent vaccine and characteristics of semen, focusing also in HPV genotype; and
- associations between HPV genotypes included in the 9-valent vaccine and miscarriage.

### Statistical Analysis

No statistical analysis was performed.

## Results

Many research teams were interested in the possible role of HPV infection in couple infertility. Articles were mainly focused on HPV infection and its relationship with infertility, semen parameters, and IVF outcomes. Only some authors discussed the relationship between HPV and fertility parameters specifying the HPV types or risks.

The selected articles concerning HPV genotypes included in the 9-valent vaccine were as follows:

- 10 of 14 articles focused on the effect of HPV on *in vitro* fertilization outcomes;
- 9 of 11 articles studied the effect of HPV on semen parameters; and
- 13 of 15 articles studied the effect of HPV infection on the risk of miscarriage.

### Prevalence of HPV Genotypes Included in the 9-Valent Vaccine in Infertile Women and ART Success

Studies examining the impact of HPV genotypes on female fertility are limited. Most studies investigate the prevalence of HPV-positive women in ART programs or the connection between HPV infection and ART success. Table 1 summarizes the literature regarding the effect of HPV on infertility and on assisted reproductive outcomes.

**Table 1:** Literature regarding the effect of HPV included in the 9-valent vaccine on infertility and assisted reproductive outcome.

**References**	**Study design**	**Results**	**Association**
Rocha et al. (18)	Case–control study (*n* = 60)	Association between HR-HPV infections (HPV 16, 31, 66, and 82) in the upper female genital tract and infertility/endometriosis.	Yes (infertility, endometriosis)
Lundqvist et al. (19)	Prospective cohort study (*n* = 214)	7% of women undergoing IVF, as opposed to 9.1% of healthy control women, tested positive for HR-HPV 16, 18, 31, 33, or undetermined. IVF treatment was successful in 66 (33%) of the 198 women and in 7 (50%) of the 14 women infected by HR-HPV (16, 18, 31, 33, or unknown type).	No
Strehler et al. (20)	Prospective controlled study (*n* = 294 vs. 2,262)	The prevalence of HR-HPVs (16, 18, 31, 33, 35, 39, 45, 51, 52, 54, 56, and 58) in the screening study (*n* = 2,262) was 8.4%, not significantly different from the prevalence in the ART patients before (7.8%) or after (6.8%) stimulation.	No
Bugge et al. (21)	Population-based cohort study (*n* = 10,595)	No association between a HR-HPV infection (13 different types of high-risk HPV genotypes: HPV 16, 18, 31, 33, 35, 39, 45, 51, 52, 56, 58, 59, and 68) and risk of female factor infertility, neither for a single HPV-positive test nor for a persistent HPV infection.	No
Spandorfer et al. (22)	Prospective study (*n* = 106)	Over 14% of all patients tested were positive for a HR-type (16, 18, 31, 33, 35, 39, 45, 51, 52, 56, 58, 59, or 68). Patients with HPV were less likely to become pregnant after undergoing IVF (23.5 vs. 57%)	Yes (ART outcome)
Depuydt et al. (23)	Retrospective analysis (*n* = 590)	HPV-positive women for HPV 6, 11, 16, 18, 31, 33, 35, 39, 45, 51, 52, 53, 56, 58, 59, 66, 67, or 68 undergoing IUI are six times less likely to become pregnant per IUI cycle than HPV-negative women (11.36 vs. 1.87%). In both single and multiple infections, HPV 16 was the most prevalent type (16.1%), followed by HPV 31 (14.8%) and HPV 53 (12.8%).	Yes (IUI outcome)
Comar et al. (24)	Prospective cohort study (*n* = 82)	15% of women were positive for HPV: genotyping analysis showed a high prevalence of HR types (92%) including two HPV 16, two HPV 58, two HPV 33, one HPV 54, one HPV 52, one HPV 60, one HPV 68, and two HPV at low risk (HPV 6). Among HPV-positive women, live birth rate was about half of the rate in HPV-negative women (not significant).	No
Tanaka et al. (25)	Observational study (*n* = 192)	Presence of the HPV type 16 in the cervix did not have any impact on IVF treatment variables.	No
Jaworek et al. (26)	Observational laboratory-based study (*n* = 207 vs. 945)	Despite the high prevalence of HR-HPV in both oocyte donors (*n* = 207) and infertile women (*n* = 945), HPV infection did not influence the outcomes of ART. HPV 16 occurred most frequently (21.4% of HPV-positive samples), and it was the most prevalent HPV genotype in infertile women treated with IVF (27.1%).	No
Yang et al. (27)	Retrospective analysis (*n* = 3,880)	HPV positivity (16, 18, 31, 33, 35, 39, 45, 51, 52, 56, 58, 59, or 68) did not appear to affect ART outcomes. HPV therapy is not recommended because delay in the IVF treatment could determine inferior ART outcomes.	No

Some studies examine the association between HPV infection and infertility. Recently, it has been underlined by Rocha et al. that many high-risk HPV infections included in the 9-valent vaccine are associated with infertility and endometriosis (18). Cobas 4800 HPV testing was used to detect HPV in three separate channels that discriminate HPV 16 individually, HPV 18 individually, and a pool of 12 other HR-HPV types (HPV 31, 33, 35, 39, 45, 51, 52, 56, 58, 59, 66, 68). In addition, it was observed that HR-HPV 16, 31, 66, and 82 had an infection continuum from the lower to the upper genital tract (18).

These results contrast with the observations by Lundqvist et al. (19) that observed that 7% of women undergoing IVF as opposed to 9.1% of healthy control women tested positive for HR-HPV 16, 18, 31, 33, or undetermined. Also, Strehler et al. (20) did not find a higher incidence of HPV infection in the infertile population (*n* = 294, age range 22–45) compared to the general population (*n* = 2,262, age-matched). Finally, Bugge et al. (21) did not find an association between HR-HPV infection (HPV 16, 18, 31, 33, 35, 39, 45, 51, 52, 56, 58, 59, and 68) and infertility.

In the prospective study of Spandorfer et al. (22), evaluating the prevalence of HPV in women undergoing IVF in North America, women with a cervical HR-HPV infection (16, 18, 31, 33, 35, 39, 45, 51, 52, 56, 58, 59, or 68) had a significantly lower number of pregnancies after ART than women with negative HPV results (23 vs. 57%). The authors found no association between HPV and atypical responses to ovarian stimulation and between HPV and tubal infertility. Overall, no differences were noted in the etiology of infertility comparing HPV-positive and HPV-negative groups. Concerning the mean age, the number of oocytes retrieved, the number of embryos transferred, and the embryo quality, no differences were found between the two groups. The need for intracytoplasmic sperm injection and the fertilization rate of mature oocytes did not differ between HPV-positive and HPV-negative women. No mechanisms linking HPV infection and negative IVF outcomes were found. However, the pregnancy rate in HPV-positive women was less than half of that observed in patients of similar age with a negative result following HPV screening.

A recent study by Depuydt et al. (23) investigated the impact of HPV on intrauterine insemination (IUI) outcome, reporting that women suffering from an HPV infection undergoing IUI treatments have lower chances to become pregnant. More in detail, among 590 patients undergoing 1,529 IUI cycles, those affected by HPV had a six times lower chance to achieve pregnancy (1.87%) compared to women not infected by HPV (11.4%). HPV 6, 11, 16, 18, 31, 33, 35, 39, 45, 51, 52, 53, 56, 58, 59, 66, 67, and 68 were tested. Low-risk HPV types were not detected. In single-type HPV infection, the most prevalent type was HPV 53 (20.5%), followed by HPV 66 (19.3%) and HPV 16 (13.3%). In infections with multiple types, the more prevalent were HPV 16 and 31 (19.7%). Overall, in both single and multiple infections, the most prevalent type was HPV 16 (16.1%) followed by HPV 31 (14.8%) and HPV 53 (12.8%). Among these, HPV 16 and 31 are included in the 9-valent vaccine. It is interesting that most women (97.3%) who underwent IUI were not vaccinated.

Comar et al. (24) found no significant differences in ovarian response and implantation rate comparing HPV-positive women to HPV-negative ones. Also, the miscarriage rate was not significantly different. However, the live birth rate was about double among HPV-negative women when compared with HPV-positive women, although the difference was not significant probably because of the low number of cases. HPV 16, 58, 33, and 6 found in infertile women are included in the 9-valent vaccine.

An investigation in Japan of 192 women undergoing IVF (25) observed that there was no relationship between HPV 16 detection and any IVF treatment variable, including the cause of infertility or IVF outcome.

Javorek et al. (26) and Yang (27) found no associations between HPV included in the 9-valent vaccine and ART outcomes despite the high prevalence of HR-RPV in both oocyte donors and infertile women. Moreover, Javorek (26) reported that there was no association between HR-HPV-positive status and abortion rate in spontaneously pregnant women.

### HPV Included in the 9-Valent Vaccine Genotypes and Semen Alterations

Table 2 summarizes the literature regarding the effect of HPV on seminal liquid.

**Table 2:** Literature regarding the effect of HPV on seminal liquid.

**References**	**Study design**	**Sperm cell count (million/ml)**	**Sperm cell motility (%)**	**Normal morphology cells (%)**	**HPV genotype**
Lai et al. (28)	Descriptive clinical study (*n* = 24)	Oligozoospermia in:33% HPV 1618% HPV 18	Considered significantly associated (control) 40.5 ± 18.6 75% HPV+, 83% HPV 16, 73% HPV 18	75.0 ± 7.6	25% HPV 16 46% HPV 18 Oligozoospermia was found in 33% and 18% specimens that were positive for HPV type 16 and 18 DNA, respectively
Bezold et al. (29)	Retrospective controlled study (*n* = 241)	Decreased 139.3 (13.0–214.5)	Statistically non-significant trend for lower total motile sperm count	–	HR-HPV (16, 18, 31, 33, 35, 39, 45, 51, 55, 53, 58, 59, 66, 68, 69, 70, and 73) LR-HPV (6, 11, 32, 44, 55, 61, 62, 74, 83, 84, and 89) 3 out of the 8 cases that tested positive for generic HPV DNA were subtyped as HPV 16
Foresta et al. (30)	Cross-sectional clinical study (*n* = 290)	Decreased 30.0 ± 21.5	Decreased 33.9 ± 15.9	32.9 ± 13.9	6, 11, 16, 18, 26, 31, 33, 35, 39, 40, 42, 45, 51, 52, 53, 54, 55, 56, 58, 59, 61, 62, 64, 66, 67, 68, 69, 70, 71, 72, 73, 81, 82, 83, 84 10.2% HPV 16
Foresta et al. (31)	Cross-sectional clinical study (*n* = 200)	57.5 ± 30.4	Decreased 37.7 ± 16.8	31.5 ± 8	6, 16, 18, 53, 58, 59, 61, 62, 66, 70 4% HPV 16 and HPV 18 4% HPV 6
Foresta et al. (32)	Cross-sectional clinical study (*n* = 32)	Decreased 32.4 ± 21.1	Decreased 29.7 ± 13.8	17.8 ± 9.1	In decreasing order of prevalence: 6, 53, 18, 16/90, 84, 61/62
Yang et al. (33)	Case–control study (*n* = 1,138; 107 infertile HPV+)	Infertile 111.31 ± 78.51 Fertile (control) 114.42 ± 61.65	Infertile: Decreased 20.5 ± 10.4 Fertile (control): Decreased 32.2 ± 10.0	Infertile: significantly decreased 4.66 ± 3.08Fertile (control): 8.51 ± 4.21	Most common genotypes in infertile men in decreasing order: 45, 16, 52, 59, 18, 33 Genotypes 45, 52, 18, 59, and 16 are significantly higher in infertile men than in fertile men Most common genotypes in fertile men in decreasing order: 68/81,33,39
Schillaci et al. (34)	Cross-sectional clinical study (*n* = 308)	Decreased 10	Decreased 30	60	16, 51, 52, 59 most frequent; also found 11, 18, 31, 33, 39, 44, 53, 61, 66, 70, 73, 83, 84, and 87 prevalence HPV 52 (21%) - 90% HR-HPV - 40% multiple infection HR-HPV - 40% concordance of all viral genotypes with female partner - 50% concordance at least of one viral genotype with female partner
Garolla et al. (35)	Cross-sectional clinical study (*n* = 35)	Decreased 32.0 ± 11.2	Decreased 29.0 ± 11.4	18.8 ± 6.2	6, 11, 16, 18, 26, 31, 33, 35, 39, 40, 42, 43, 44, 45, 51, 52, 53, 54, 56, 58, 59, 66, 68, 69/71, 70, 73, 74, 82
Moghimi et al. (36)	Case–control study (*n* = 140)	51.38 ± 29.29	Decreased 23.5 ± 13.5	7.13 ± 2.64	HR-HPV 16, 18, 31, 33, 35, 39, 45, 51, 52, 56, 58, 59

All studies related to the correlation between male fertility and HPV, including also those excluded because the HPV genotype was not specified, reporting a clear correlation between HPV and asthenospermia and infertility.

Some authors decided to examine the result for specific genotypes. Lai et al. (28) found an association between HPV and oligoasthenozoospermia, but, when considering oligozoospermia, it was found in 33 and 18% of sperm specimens that were positive respectively for HPV 16 and 18. Similarly, they investigated the correlation between genotypes 16 and 18 and asthenozoospermia, finding out that it was present in 83% of men with HPV 16 infection and in 73% of patients with HPV 18 infection.

In addition, Bezold et al. (29) wanted to clarify the percentage of infertile men with HPV 16 infection, finding a prevalence of 37.5%. Moreover, it is interesting to note that the test used to search HPV DNA in that study identified almost all the HR-HPV and all the genotypes included in the 9-valent vaccine.

Similar results have been found by Foresta et al. (30) and Garolla et al. (35): the searched genotypes are present in the 9-valent vaccine. Moreover, Foresta found a relevant percentage of cases (10.2%) positive for HPV 16. In a recent study conducted by Moghimi et al. (36), all the HR-HPV types present in the 9-valent vaccine were detected in the positive population. In another study, Foresta et al. (31) used a more specific test identifying fewer HPV genotypes (6, 16, 18, 53, 58, 59, 61, 62, 66, and 70); however, they wanted to clarify that 4% were HPV 16 and HPV 18 positive, while 4% were HPV 6 positive. In a more recent study (30), the authors specified what genotype was found in order of prevalence: HPV genotypes related to infertility were 6, 53, 18, 16/90, 84, and 61/62. Yang et al. (33) reported that the most common HPV types in decreasing order of prevalence in infertile men were HPV 45, 16, 52, 18/59, and 33; in fertile patients, the most common genotypes significantly differ: HPV-68/81, −33 and −39. A study published in 2013 (34) revealed that 90% of infertile patients were affected by HR-HPV infection. Another study wanted to determine the HR-HPV infection rate among couples undergoing IVF, revealing that HPV 52 was the most commonly found in the case of semen alteration (37), while other studies that specifically targeted oncogenic genotypes found HPV 52 (38) and HPV 16 (39) as the most common genotype overall in semen samples. Other researchers (11, 26) reported that HPV 16, HPV 51, HPV 52, and HPV 45 are often found in semen.

### HPV Genotypes Included in the 9-Valent Vaccine Infection and Risk of Miscarriage

Table 3 summarizes the literature regarding the effect of HPV genotypes included in the 9-valent vaccine on the risk of miscarriage: a possible association is reported in a few studies (38, 40, 41) while many articles reported no association with miscarriage (16, 17, 22–24, 26, 42–45).

**Table 3:** Literature regarding the effect of HPV genotypes included in the 9-valent vaccine on the risk of miscarriage.

**Study and year**	**Study design**	**Results**	**Association**
Hermonat et al. (40)	Prospective cohort study (*n* = 40)	60% of spontaneous samples were found to be positive for HPV types 6, 11, 16, and 18. In comparison, only 20% of elective abortion samples were positive.	Yes
Sikström et al. (41)	Cross-sectional study (*n* = 66 vs. 900)	12% of spontaneous abortion in 66 women with current genital HPV infection (HPV types 6, 11, 16, 18, 31, 33, and 35) (12%) compared with 6% of abortion in HPV-negative women, despite a lower anamnestic rate of pregnancies in HPV-positive women.	Yes
Perino et al. (38)	Prospective study (*n* = 199)	All IVF pregnancies in HPV-positive couples resulted in miscarriage. The most frequent genotypes involved were HPV 16 and HPV 66, whereas HPV 51 and HPV 52 were most frequently identified in the male partner of infertile couples.	Yes
Spandorfer et al. (22)	Prospective study (*n* = 106)	No differences in spontaneous abortion rates were noted between HPV-positive (16, 18, 31, 33, 35, 39, 45, 51, 52, 56, 58, 59, or 68) (*n* = 17) and HPV-negative (*n* = 89) patients.	No
Depuydt et al. (23)	Retrospective analysis (*n* = 590)	No association found between spontaneous abortion and HPV cervical infection (HPV tested: 6, 11, 16, 18, 31, 33, 35, 39, 45, 51, 52, 53, 56, 58, 59, 66, 67, and 68).	No
Ticconi et al. (42)	Retrospective case–control study (*n* = 524)	Women with recurrent miscarriage have a lower prevalence of HPV DNA tests (HPV 16, 18, 31, 33, 35, 39, 45, 51, 52, 56, 58, 59, or 68) than controls. This suggests that immune reactivity potentially leading to recurrent miscarriage could be in some way protective against genital HPV infection.	No
Comar et al. (24)	Prospective cohort study (*n* = 82)	Miscarriage rate was not significantly different between the HPV-positive IVF patients and HPV-negative ones.	No
Skoczynski et al. (43)	Cross-sectional study (*n* = 51)	HPV 16/18 infection rate does not seem to be higher in cases of spontaneous abortions.	No
Jaworek et al. (26)	Observational laboratory-based study (*n* = 207 vs. 945)	No association between high-risk HPV infection and higher miscarriage risk were found.	No
Yang et al. (44)	Retrospective analysis (*n* = 3,880)	In pregnancy cases after IVF treatments, couples with positive HPV test in male partners had a significantly higher spontaneous abortion rate compared with the HPV-negative group (66.7 vs. 15.0%). HPV infection in females could also increase this rate, but without statistical significance (40.0 vs. 13.7%).	No
Conde-Ferraez et al. (45)	Case–control study (*n* = 281)	HPV cervical infections (HPV types 16, 18, and 58, and low-risk types 6/11) were not associated with spontaneous abortion.	No
Henneberg et al. (17)	Experimental model	Increased DNA fragmentation and trophoblastic death in blastocysts in HPV 16 and 18 positive (mice).	Yes
Calinisan et al. (16)	Experimental model	Increased DNA fragmentation and trophoblastic death in blastocysts in HPV 16, 18, 31, or 33 positive (mice).	Yes

As early as 1997 (40), it has been shown that HPV infection (types 6, 11, 16, and 18) was three times more prevalent in spontaneous abortion specimens compared with elective ones (60 vs. 20%, respectively) opening the door to the hypothesis that HPV could be an etiologic agent of some miscarriages. Moreover, it was demonstrated that these viruses might be closely linked to fetal pathology.

In 1995, a study by Sikström et al. (41) recorded a two-fold increase in the rate of spontaneous abortion in 66 women with current genital HPV (types 6, 11, 16, 18, 31, 33, and 35) infection (12%) compared with 900 HPV-negative women (6%), despite a lower anamnestic rate of pregnancies in HPV-positive women.

Perino et al. (38) stated that couples who underwent ART cycles experienced an increased risk of pregnancy loss when HPV DNA testing was positive in the male partner, compared with non-infected patients (66.7–15%, *p* < 0.001). Moreover, all pregnancies in HPV-positive couples resulted in miscarriage, whereas there was a 15.9% overall miscarriage rate in HPV-negative couples (*p* < 0.001). The most frequent genotypes in women were HPV 16 and HPV 66, whereas HPV 51 and HPV 52 were the most frequently identified in the male partner of infertile couples. HPV 16 and 52 are included in the 9-valent vaccine showing protection for women, male partners, and infertile couples.

A study in 2013 (42) stated that women with recurrent abortion have a lower prevalence of HPV DNA test positivity than controls, suggesting that immune reactivity potentially leading to recurrent miscarriage could be protective against genital HPV infection. However, the study was confined to women with recurrent miscarriages. Other researchers claimed that there is no correlation between women's HPV infection and miscarriage rate at all (24, 26, 27).

## Discussion

Infertility affects millions of people, about 10–30% of reproductive-age couples all over the world (1, 2). At the same time, HPV infection is one of the most common STD with high prevalence in some developing and underdeveloped countries, and its consequences cannot be underestimated (4, 5). HPV vaccination remains unavailable in many countries due to economic issues and low public acceptance. The purpose of this comprehensive review is to research the available knowledge regarding the implication of HPV genotypes included in the 9-valent vaccine HR-HPV 16, 18, 31, 33, 45, 52, and 58 and low-risk HPV (LR-HPV) 6 and 11 in couple infertility, with the aim to hypothesize if vaccination could have a protective role for reproductive health, eventually improving vaccine acceptance.

It is interesting to note that many studies reported the involvement of genotypes not included in the 9-valent vaccine. These HPV genotypes may have a role in infertility though they are not yet part of the ones contained in the vaccine. Moreover, the list of known HR-HPVs could change in the next years as the genotypes contained in the vaccine. Arbyn et al. (46, 47) suggested increasing the HR-HPVs in the list from 14 up to 20 due to the growing carcinogenicity of potential/possible risk types. The phenomenon of growing carcinogenicity, including the ability of constant evolution of HPV types, should be considered (48). In IVF patients, a Pap smear is required before the stimulation cycle. In case of abnormal results, further analysis must be conducted, including a high-risk HPV test and/or colposcopy and pathology biopsy. Patients with cervical pathology higher than cervical intraepithelial neoplasia-1 (CIN-1) cannot be enrolled in IVF cycles.

Studies that evaluated the impact of HPV on ART outcomes have shown conflicting results. Most studies did not find a higher incidence of high-risk HPV infections included in the 9-valent vaccine in the infertile population (19–21, 24–27). However, we noticed some limitations of these studies. Lundqvist et al. (19) did not apply age limits: the case and control groups were 20–40 and 25–59 years old, respectively. Moreover, both case and control groups were substantially smaller compared with the previously cited study (18). These criticisms cannot be applied to the study of Strehler et al. (20). However, it is interesting to note that all patients with prior cervical surgery, cervical dysplasia, or a HR-HPV infection were excluded from the study group. Bugge et al. (21) found that the prevalence of high-grade cervical lesions was twice as high in women with infertility compared with the general population, implying a more complex association between HPV and infertility. The study of Tanaka et al. (25) was not conducted on a large population (10 women and 4 men were found to be HPV type 16 DNA positive). Finally, Javorek et al. (26) found that HPV 16 occurred most frequently (21.4% of HPV-positive samples) in infertile women treated with IVF, and it was the most prevalent HPV genotype (27.1%, 13/48).

One of the most quoted articles on the subject is from Spandorfer et al. (22), finding a significantly lower number of pregnancies after ART in women with HPV infection than in women with negative HPV results. The presence of high-risk HPV types probably indicates that these patients are unable to spontaneously clear these viruses. They speculated that immunologic conditions that reduce the likelihood of spontaneous HPV clearance also may limit the likelihood of embryo implantation. In fact, an innate or acquired decrease in the ability to generate the production of high levels of proinflammatory cytokines would explain the linking between HPV infection and lower success of IVF. In addition, Depuydt et al. (23) reported a connection between HPV infection and infertility, but the population study was limited to women who underwent the IUI technique.

Also, Rocha et al. (18) reported an association between HR-HPV infertility and infections, observing an association with endometriosis. Three previous studies have evaluated the possible correlation between endometriosis and HPV (49–51). Two of these (49, 51) highlighted the importance of the high-risk HPV infection from the lower genital tract to the upper genital tract sites. More specifically, the association between endometriosis and viral STD infections has been investigated by Oppelt et al. (49) using PCR-based enzyme-linked immunosorbent assay in 66 tissues, including peritoneum, endometrium, and ovary. HR-HPV included in the 9-valent vaccine (16 or 18, and possibly one among HPV 31, 33, 35, 39, 45, 51, 52, 56, 58, 59, or 68) were detected in 11.3 and 27.5% of lesions in the case and control groups, respectively. Heidarpour et al. (51) found an association between the presence of HR-HPV types (16, 18, 31, 33, 35, 39, 45, 52, 56, 58, and 59) in the upper genital tract and infertility, primarily caused by endometriosis. Conversely, Vestergaard et al. (50) searched the presence of HPV in endometriotic samples using sensitive PCR tests, finding a low prevalence of HPV 35, 68, 70, and 90 in endometriotic lesions. They concluded that HPV could not be the cause of endometriosis. However, no genotypes are included in the 9-valent vaccine.

In conclusion, the effect of HPV infection in women on ART outcome remains undefined also due to the methodological limitations of the studies performed. However, for years now, it has been known to clinicians studying reproductive medicine that the patient must always be the couple and not the individual. It is precisely in this consideration that we could attempt to explain the conflicting data. Most studies analyze the connection between HPV and infertility or ART outcome searching women HPV infection. However, taking the assumption that most couples suffering from infertility are stable couples, we should consider both partners positive for HPV regardless of whether the detection of the virus is done on a sperm or cervical swab. We should take into consideration HPV positivity not only in women but also in men. If we correlate infertility only with cervical positivity for HPV, we could not consider a large part of HPV-positive couples. On the other hand, any molecular techniques for the detection of HPV including PCR do not have a 100% sensibility. Not by chance, if we analyze the connection between ART and HPV by only studying sperm infections, the results will agree. Therefore, we should analyze the impact of HPV in infertility and ART outcomes by studying the presence of HPV not only in the female genital tract but also in the male counterpart.

In the past years, some viral diseases such as hepatitis B and HIV were widely studied in men attempting ART to reduce the risk of transmission of STDs via the seminal fluid. Nevertheless, HPV infection was not considered as well.

In the last years, there has been an increasing interest in the prevalence of HPV among men. Many studies have provided evidence of possible HPV-related subfertility in men, suggesting that HPV infection could be a risk factor (36). Indeed, HPV has been isolated in seminal samples, and its presence has been correlated with idiopathic asthenospermia (28, 31, 52, 53), sperm cell numbers (29), and semen pH (54). All studies related to the correlation between male fertility and HPV, including also those excluded because the HPV genotype was not specified, reporting a clear correlation between HPV and asthenospermia and infertility.

However, not only HPV detection but also HPV genotyping could be of great value in infertility diagnosis at least in idiopathic infertility cases. As for the risk of carcinogenesis, another classification of HPV regarding the risk of fertility alteration may be considered after in-depth investigations (10). That is why some authors decided to examine the result for specific genotypes.

It is interesting to note that many HR-HPV genotypes included in the 9-valent vaccine have a close relationship with semen alterations. Nevertheless, the role of HPV in determining couple infertility cannot be relegated to the mere damage to sperm production. Indeed, infected seminal liquid could be a carrier of HPV DNA in the reproductive tract, with the possibility of detrimental effects on oocytes during fertilization (55, 56). Men with infected sperm should be adequately counseled before an IVF treatment (32).

The results of the study conducted by Perino et al. (38) showed, for the first time, a significant increase in the risk of abortion if sperm cells of the male partner are infected by HPV. In addition, all the pregnancies achieved in couples where both partners were HPV positive resulted in a pregnancy loss.

A recent study by Garolla et al. (57) investigated 226 infertile couples, finding out that HPV-positive couples with infertility problems had a significant reduction of cumulative pregnancy rate by ART in both IUI (from 20% in HPV negative to 9.5% in HPV positive) and ICSI (from 40.8% in HPV negative to 18.2 HPV positive). However, the prevalence of HPV in couples was determined by semen analysis instead of cervical analysis, and HPV genotypes were not identified.

While it is widely recognized that spontaneous abortion can be linked to fetal genetic abnormalities or viral infections involving cytomegalovirus (58), Epstein–Barr virus (59), Herpes Simplex (60), mumps (61), and rubella (62), little is known about HPV and early embryos.

The association between HPV infection and failed placental invasion has already been widely demonstrated in studies that correlate HPV infection with spontaneous preterm delivery (63). The HPV-transfected blastocysts could have a progressive loss of invasiveness leading to spontaneous abortion, before pregnancy diagnosis. Studying the connection between HPV infection and miscarriages after pregnancy has been difficult.

Conde-Ferráez et al. (45) did not detect an association between HPV 16, 18, 58, 6, and 11 and spontaneous abortion, but they found out that HPV prevalence tends to increase during the trimesters of pregnancy. They speculated that it could be caused by elevated estrogen levels that may affect viral replication or alter immunity during pregnancy. This suggests the difficulty in finding the connection between miscarriage and the real prevalence of infection in the couple if we analyze only cervical samples. The immune capacity of resolving the HPV infection and the lack of some HPV positivity in those women who contracted the infection from their partner after the cervical withdrawal make it difficult to determine the real HPV prevalence and consequently the miscarriage correlation. In addition, Ticconi et al. (42) and Spandorfer et al. (22) suggested that immune reactivity, potentially leading to recurrent miscarriage, could be, in some way, protective against genital HPV infection.

Some studies (22, 23) did not find a correlation between HPV infection and miscarriage but noticed a higher failure of ART. Although it is very difficult to discriminate between an initial pregnancy loss and a reduced ART pregnancy rate, we could hypothesize that mechanisms involved in ART failure could be the same ones causing miscarriage in other studies.

In 1997, Hermonat et al. (40) noticed a high prevalence of HPV infection in spontaneous abortion samples; however, he did not hypothesize any etiopathogenetic cause. In the latest studies, both Sikström et al. (41) and Hermonat (40) explained the cause of a higher rate of abortion with the possible transmission of the virus to oocytes during fertilization, which affects or induces the immune system response.

A recent review of Isaguliants et al. (64) assumed that infertility may be associated with an anti-HPV immune response. Immune system activation to eliminate HPV-infected cells could cause an immune rejection of the HPV-infected embryo as a maternal graft-vs.-host disease against HPV-infected fetuses.

The same authors underline that HPV infection could arrest the aquaporin AQP8 function involved in the cleaning of excessive reactive oxygen species, causing additional oxidative stress in sperm and oocyte, resulting in additional DNA damage and apoptosis (65, 66). Moreover, HPV infection can reduce the cells' ability to repair DNA, amplifying its damage on gonadal cells (67).

*In vitro* experiments (17) have shown that trophoblast cells in women with HPV 16 or 18 infection have a higher chance of stage-specific maturation arrest and apoptosis and a reduced placental invasion into the uterine wall compared with control cells. The same studies showed that HPV exposure and two-cell embryo demise were associated. Furthermore, the exposure of HPV until later embryo stages causes some deleterious effects on embryos. HPV 16 decreases blastocyst formation, while HPV 18 inhibits the blastocyst hatching process (17).

In a study by Foresta et al. (68), the oocytes penetrated by transfected sperm (hamster egg penetration test) with human HPV 16 expressed the viral gene, suggesting an active transcription mechanism. To confirm these data, *in vivo* studies should be performed.

Although the precise pathway that the virus uses to infect sperm cells has not been identified, Perez-Andino et al. (69) showed that HPV (type 16, 18, 31, or 33) capsid binds to two distinct sites at the equatorial region of the sperm head surface. This suggests that sperm cells promote virus dispersal and mucosal penetration within the female genital tract. Many studies demonstrated that the relationship between HPV and miscarriage could be explained by the transmission of virus-destabilized genes to oocytes during fertilization, determining apoptosis of the embryo cells through DNA fragmentation (16, 17, 56). When the HPV virus infects sperm liquid, it could affect the acrosomal reaction, leading to a reduction of the acrosome capacity and functionality. Fujita et al. (70) suggested that this could be related to the presence of E6–E7-derived HPV DNA genes associated with cell transformation. In particular, the expressed oncoprotein of the HPV E6 gene degrades p53 protein through the cellular ubiquitin-protein ligase E6–AP pathway (71, 72). At the same time, E7 oncoprotein binds to the retinoblastoma gene products, pRb, p107, cyclin A, AP-1 transcription factor, and the TATA box binding protein, TBP 58-60. This leads to cellular instability. The sites for HPV DNA integration in the host genome seem to be near oncogenes (73). This would determine the transformation of cells at the chromosomal fragile site (74, 75), at 3p14 (76, 77), and the FRA8C site or at 8q24 (78), causing a gene interruption and a loss of chromosome heterozygosity (79).

This topic is relevant because HPV has a very high prevalence (65%) among men aged 18–40 years (80). It is estimated that 10% of male subjects may have a subclinical infection during an extended period of their life (29, 31). Moreover, sperm washing in preparing and purification of seminal liquid used in ART is not able to remove the risk of transmission of viral infection (81, 82), thus allowing viral transmission through artificial insemination procedures (54, 82, 83).

The literature on the association between HPV infection and abortion is still conflicting. Few older prospective or cross-sectional studies and “*in vitro*” studies have shown a possible relation, especially with high-risk viral strains. However, most recent clinical studies did not show a possible association. Considering the results of the experimental studies and the strong association between HPV infection and male infertility, we could hypothesize that infected spermatozoa may play a role as carriers of HPV DNA both in the reproductive tract and within the oocyte, with the possibility of detrimental effects during fertilization. This suggests to us the need for further studies concerning the association between HPV infection and miscarriage, testing contemporary HPV DNA in female and male counterparts.

In conclusion, while numerous reviews analyzing the correlation between HPV and male infertility seem to find agreement (84–87), there are few analyses that speculate about the correlation between HPV and female or couple infertility (64, 88). The few reviews available do not seem to find agreement with each other and with many other published studies. The conflicting results of some of these can be explained by the disjointed analysis. Our review focused in particular on the viral genotypes included in the 9-valent vaccine. Few studies consider the impact of viral infection on both partners and the product of conception, and none have focused on the genotypes included in the vaccine. We believe that it is necessary to search HPV infection in both female and male partners and to genotype HPV to discover the real impact of HPV infection on the risk of infertility or ART failure.

The relevance of the problem raised in this paper is undeniable, given how many HPV-infected couples, many of whom are also infertile as a couple, try to solve the problem of childbirth every day. In general, the value of vaccination against cervical cancer has been well-proven globally. Now, researchers should shed light on the issues of additional benefits that vaccination might be able or not able to provide.

## Conclusions

To date, there is no agreement in the literature on the implication that HPV infection has on the fertility and miscarriage rate. Although it can be stated that HPV prevalence among couples with infertility problems undergoing IVF treatment is consistent, it does not seem to affect the performance of oocytes. Otherwise, HPV infection affects sperm parameters, in particular spermatozoa motility.

The proven correlation between HPV male infection both with infertility and abortion should implement new gynecological researches: it appears always clearer that it is necessary to consider contemporary female and male infection, particularly for infertile couples.

The HPV DNA testing in male partners of infertile couples could be useful to follow up individuals with infected sperm. In these cases, despite the absence of treatment for HPV infection, the possibility of delaying IVF procedures until the viral infection has been cleared or eliminating HPV from sperm through a specific washing procedure of semen (89) could be considered. HPV genotype could be useful for counseling and the decision-making process.

The correlation between HPV male infection both with asthenozoospermia and increased risk of pregnancy loss could recommend the extension of anti-HPV vaccination to adolescent males, along with anogenital and oral cancer prevention. In fact, when an association can be found, most HR-HPV involved are those included in the 9-valent vaccine.

Despite the fact that the relation between 9-valent HPV genotypes involved in female infection and miscarriage/infertility is not clear, the impact of this virus on health reproduction is evident. Considering this, the importance of HPV vaccination in adolescent females is confirmed for not only preventing cancer but also couple infertility.

A vaccine efficacy study could be useful to confirm the importance of primary prevention for the reproductive health of couples.

## Key Questions

➢ **Can HPV infection have a role in female infertility?** To date, most studies did not find an association between female infertility and HPV infection.
➢ **Can HPV have a role in ART outcomes?** To date, it is not in accordance with the literature. Most included studies did not find a possible association between HPV women infection and ART outcomes.
➢ **Can HPV infection have a role in male infertility?** All included studies showed an association between asthenozoospermia and HPV male infection.
➢ **Can HPV infection modify the risk of miscarriage?** To date, there is no agreement in the literature. Further prospective studies are necessary to confirm this association. We believe that it is necessary to search HPV infection in both females and males and to genotype HPV to discover the real impact of HPV infection on the risk of miscarriage.
➢ **Can immunological status modify the risk of infertility in couples affected by HPV?** Immune system, pregnancy, and HPV infection appear to interact by modifying the host's immune response. The capacity of resolving the HPV infection in those women that contracted the infection from their partner makes it difficult to determine the real HPV prevalence and consequently the miscarriage correlation.
➢ **Can HPV the 9-valent vaccine have a role in preventing infertility?** To date, we could hypothesize that the 9-valent vaccine could have an important role in preventing male infertility because current literature shows a correlation with HPV genotypes included in the 9-valent vaccine.

## Author Contributions

AC and JDG contributed to conception and design of the study. LG and GDC organized the database. CM, JDG, and FS wrote the first draft of the manuscript. All authors contributed to manuscript revision, read, and approved the submitted version.

## Conflict of Interest

The authors declare that the research was conducted in the absence of any commercial or financial relationships that could be construed as a potential conflict of interest.

## Publisher's Note

All claims expressed in this article are solely those of the authors and do not necessarily represent those of their affiliated organizations, or those of the publisher, the editors and the reviewers. Any product that may be evaluated in this article, or claim that may be made by its manufacturer, is not guaranteed or endorsed by the publisher.

## Abbreviations

ACOG: American College of Obstetricians and Gynaecologists
STDs: sexually transmitted diseases
PID: pelvic inflammatory disease
TFI: tubal factor infertility
HPV: human papilloma virus
HR-HPV: high-risk HPV
ART: assisted reproductive technology
LR-HPV: low-risk HPV
CIN-1: cervical intraepithelial neoplasia-1
IVF: *in vitro* fertilization.
